# Presence and Impact of Aldol Condensation Products as Off-Notes in Plant-Based Protein Sources

**DOI:** 10.1021/acs.jafc.4c10526

**Published:** 2025-03-03

**Authors:** Chloe V. Mayo, Dimitris P. Balagiannis, Valentina Stojceska, Jane K. Parker, George R. Fern

**Affiliations:** † Department of Chemical Engineering, Wolfson Centre for Sustainable Materials Development and Processing, Brunel University of London, Kingston Lane, Uxbridge UB8 3PH, U.K.; ‡ Department of Food and Nutritional Sciences, University of Reading, Whiteknights, Reading RG6 6DZ, U.K.; § Institute for Energy Future, Centre for Sustainable Energy in Food Supply Chain, Brunel University of London, Uxbridge UB8 3PH, U.K.

**Keywords:** off-notes, pea protein isolate, GC–olfactometry, plant-based meat analogues

## Abstract

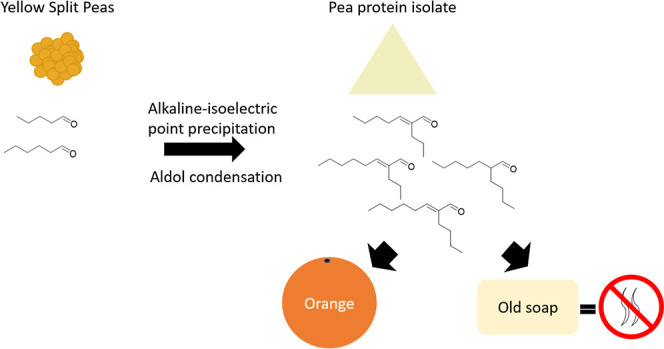

Off-notes in plant-based sources of protein are mainly formed via the lipid oxidation of unsaturated fatty acids. During gas chromatography–olfactometry analysis of pea protein isolate, previously uncharacterized old soap odors were detected. These were found to arise from a family of α,β-unsaturated aldehydes formed from the aldol condensation of pentanal and hexanal during the protein extraction process. These compounds were synthesized, and it was confirmed that they are highly odor-active and contribute to the old soap odor in pea protein isolates at very low concentrations. Comparison with rice, soy, and hemp protein isolates showed that they all contained at least one such aldol condensate, whereas they were not detected in whey protein. We suggest that the main factor determining the formation of these compounds is the manufacturing process used to isolate and dry the pea protein biomass.

## Introduction

Plant-based meat analogues (PBMAs) have risen in popularity as consumers try to reduce their meat intake, predominantly for health and sustainability reasons.^1,2^ PBMAs are not widely accepted by many meat-eating consumers due to their poor taste and texture.^3,4^ Off-notes in plant-based proteins have been linked with compounds produced during the lipid oxidation process of linoleic and linolenic acids.^5^ Naturally occurring lipases in plants hydrolyze triglycerides to less stable free fatty acids^6^ which readily oxidize to form lipid oxidation products.^7^ These compounds are produced both during processing and during storage.^8^ Lipid-derived compounds such as hexanal, (*Z*)-3-hexenal, 2-pentylfuran, 2-(1-pentenyl)furan, and 1-penten-3-one have been reported to impart grassy, beany, or green off-flavors in peas and pea protein.^9^ However, other off-notes with characteristic pea and bean aromas have been attributed to the presence of 3-alkyl-2-methoxypyrazines^10,11^ which are derived from other biosynthetic pathways.^12^ In peas specifically, the characteristic green pea odors have been attributed to 3-isopropyl-2-methoxypyrazine, 3-secbutyl-2-methoxypyrazine, and 3-isobutyl-2-methoxypyrazine.^10,11^

In addition to these green notes, other off-notes in peas are reported as sweaty (3-methyl-1-butanol), cheesy (3-methylbutanoic acid), musty (acetophenone, 1-octen-3-one), earthy (1-octen-3-one), and fatty (2,4-heptadienal, 2,4-decadienal).^13,14^ Nonbeany aroma compounds in certain binary combinations have been reported to generate beany off-notes, such as 1-octen-3-one (10 ppm) with hexanal, as well as the combination of (*E*)-2-octenal (10 ppm) and (*E,E*)-2,4-decadienal when the concentration of (*E,E*)-2,4-decadienal is ≤ 100 ppm.^13^ It has been previously reported that during the extraction and processing of protein isolates, there is a change in odor-active volatiles. Throughout the extraction process, concentrations of hexanal, 1-octen-3-ol, 3-isopropyl-(5 or 6)-methyl-2-methoxypyrazine, and 2,4-decadienal remained unchanged;^15^ however, an overall decrease in alcohols, aldehydes, and ketones was seen during the solubilization step of the process.^16^ More specifically, 2-octanol and butanoic acid have been reported to decrease below detection limits during extraction.^17^ Moreover, some compounds such as methional, benzeneacetaldehyde, and (*E*,*E*)-3,5-octadien-2-one have been reported to be formed during the isolation process.^15^ This is due to lipid degradation as well as the Maillard reaction.^18^

For this study, while carrying out aroma profiling of pea protein isolate, several previously uncharacterized off-notes were found during gas chromatography–olfactometry (GC–O). These off-notes had an old soap, green, and floral aroma, which is characteristic of pea protein isolate, as well as PBMAs. The aim of this study was to characterize these off-notes and to determine their occurrence in other plant-based proteins.

## Materials and Methods

### Materials

A commercial pea protein isolate (PPI) was purchased from Peak Supps (Bridgend, UK), and pea protein concentrates were obtained from two different commercial sources (PPC1 and PPC2). All other plant-based protein powders (hemp protein (HPI), rice protein (RPI), and soy protein (SPI)) were purchased from My Protein (Northwich, UK), and whey protein (WPI) was obtained from Volac (Royston, UK). The dried yellow split peas (YSP) were purchased from a local supermarket (Asda, UK).

### Chemicals

The following aroma standards were obtained from Sigma-Aldrich (Gillingham, UK): 2-butyl-2-octenal >95% (which was determined to be ∼96% (*E*)-2-butyl-2-octenal via ^1^H NMR) (CAS RN 13019-16-4), hexanal >97% (CAS RN 66-25-1), pentanal 97% (CAS RN 110-62-3), methional >95% (CAS RN 3268–49-3), 1-octen-3-one >95% (CAS RN 4312-99-6), (*E*)-2-octenal >95% (CAS RN 2548-87-0), dimethyl trisulfide >95% (CAS RN 3658-80-8), nonanal >95% (CAS RN 124-19-6), 1-octanol >97% (CAS RN 111-87-5), acetophenone >99% (CAS RN 98-86-2), 2-nonanone 99% (CAS RN 821-55-6), 2-ethyl-1-hexanol >99% (CAS RN 104-76-7), (*E*)-2-decenal 97% (CAS RN 3913-81-3), and redistilled diethyl ether >99.5% (CAS RN 60-29-7). Potassium hydroxide (CAS RN-1310-58-3) and ethanol 99% (CAS RN 64-17-5) were obtained from Fisher Scientific (Loughborough, UK). Saturated alkane standards were also purchased from Sigma-Aldrich. 3-Methyl-2-butene-1-thiol (CAS RN 5287-45-6) was purchased as a capsule from FlavorActiv (Thame, UK).

### Gas Chromatography–Olfactometry

Each protein powder was mixed with deionized water (40:60 ratio), and 2 g was placed inside a solid-phase microextraction vial. Each vial was placed in a water bath at 60 °C for 20 min, and then a Supelco 50/30 μm divinylbenzene/carboxen on polydimethylsiloxane SPME fiber (1 cm) (Sigma-Aldrich, Bellefonte, PA, USA) was exposed to the volatiles for a further 20 min at 60 °C.

GC–O analyses were performed on a HP 5890 Series II GC instrument equipped with a flame ionization detector (FID, Hewlett-Packard, Waldbronn, Baden-Württemberg, Germany) and an ODO II odor port (SGE, Ringwood, Victoria, Australia). A nonpolar DB-5 column (30 m × 0.25 mm × 1 μm film thickness, Agilent Technologies, Santa Clara, CA, USA) and a polar Stabilwax column (30 m × 0.25 mm × 0.25 μm film thickness, Restek, London, UK) were used, and the oven temperature was programmed from 35 to 300 °C at 8 °C min^–1^ for the DB-5 column and from 35 to 250 °C at 4 °C min^–1^ for the Stabilwax column. A slow ramp of 2 °C min^–1^ was also used on the DB-5 column to ensure good separation of the two stereoisomers of the aldol condensation products (aldol condensates). For all SPME samples, an SPME liner was used. For direct injection, a 1 μL aliquot was manually injected into a splitless liner. The carrier gas used was helium, at a rate of 2.0 mL min^–1^. The effluent from the column was split equally between the FID and the odor port. At the odor port, assessors recorded descriptions of each odor. All assessors were experienced with GC–O analysis and completed at least two runs of extruded pea protein to familiarize themselves with the product and to agree upon descriptors for common compounds. For pea protein isolate, a total of 6 assessors fully characterized the aroma profile in duplicate, whereas for all other proteins, 3 assessors smelled the appropriate region for the elution of the target aldol condensates. The detection frequency was calculated for each compound, and those below a detection frequency of 4 were not reported other than those that were of particular interest because they were similar in aroma and chemical structure to 2-butyl-2-octenal.

### Gas Chromatography–Mass Spectrometry

Preliminary studies showed that the compounds of interest were below instrumental detection limits of SPME–gas chromatography–mass spectrometry (GC–MS), therefore, a more exhaustive extraction technique was required to semiquantitate the compounds of interest. Powdered proteins (10 g) and 90 mL of deionized water were mixed using an immersion blender and placed into a 250 mL dynamic headspace flask with a Dreschel head. The flasks were placed in a water bath at 60 °C, and the heads of the flasks were connected to a preconditioned glass trap (4 mm i.d., 6 mm o.d. x 3.5 mm long), packed with Tenax TA (Supelco, Poole, United Kingdom), and the system was swept with nitrogen gas at 40 mL min^–1^ for 1 h. An internal standard of 1,2-dichlorobenzene (1 μL at 100 mg L^–1^) was added to each trap, and then 100 mL min^–1^ of nitrogen was blown through the traps for 10 min to remove excess moisture. GC–MS analysis of each protein source was carried out in triplicate.

GC–MS analyses were performed on an Agilent 7890A GC coupled to an Agilent 5975C inert XL EI/CI MSD triple axis MS (Agilent Technologies, Santa Clara, CA, USA), connected to an automated thermal desorption unit (TurboMatrix ATD, PerkinElmer, Beaconsfield, UK). A nonpolar DB-5 column (30 m × 0.25 mm × 1 μm film thickness, Agilent Technologies, Santa Clara, CA, USA) and a polar Stabilwax column (30 m × 0.25 mm × 0.25 μm film thickness, Restek, London, UK) were used.

The aldol condensates were quantitated in the protein extracts using standard addition by adding 15 μL of the authentic standard of (*E*)-2-butyl-2-octenal diluted in diethyl ether at 0, 2, 5, 15, 45, and 135 μg L^–1^ to PPC2 (10 g) which contained the lowest concentration of standard and deionized water (90 g). The mixture was vortexed and allowed to equilibrate at room temperature for 1 h, extracted by dynamic headspace as above, and analyzed as above. Each extraction was carried out in duplicate, and then the calibration curve constructed (*R*^2^ = 0.997).

C6–C25 *n*-alkanes were analyzed under the same conditions as those of the samples and standards to obtain the linear retention index (LRI) of each compound. Volatiles were identified by comparing their mass spectra and LRIs with those of authentic compounds or those reported by NIST (NIST2020.L spectra library and NIST Chemical WebBook LRI collection).

### Synthesis of Aldol Condensates

**Figure 1:**
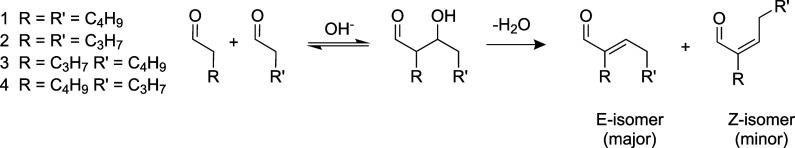
Base-catalyzed aldol condensation of pentanal and hexanal.

The aldehydes (200 μL of each aldehyde in the mixed aldol reactions or 400 μL of a single aldehyde), 2 mL of ethanol, and 2 mL of 2 M potassium hydroxide were added to a vial, vortexed (Heidolph, Schwabach, Germany) at room temperature for 15 min, and immediately cooled. Ice-cooled ethanol was added (4 mL) to ensure sufficient volume for the rotary evaporator. Individual reactions were performed with either hexanal or pentanal to form the single aldol condensates, 2-butyl-2-octenal (**1**) and 2-propyl-2-heptenal (**2**), respectively. The mixed aldol condensation reaction using both hexanal and pentanal produced a mixture containing the single aldol condensates (**1** and **2**) and the mixed aldol condensates (2-propyl-2-octenal (**3**) and 2-butyl-2-heptenal (**4**)), all as a mixture of *E*/*Z* isomers, where the *E* isomers were significantly more abundant (as discussed later) (Figure 1).

The single hexanal aldol condensates were separated from their starting materials by using a rotary evaporator. The water bath was set to 45 °C, and the vacuum was set to 175 mbar; these conditions were chosen to ensure all starting materials were separated from the sample. The sample was retained in the flask, while hexanal, hexanoic acid, and ethanol were removed into the solvent trap of the rotary evaporator. Following the rotary evaporator, an excess of diethyl ether and distilled water were added to the solution and transferred to a separating funnel and shaken. The solution was allowed to settle for 1 h; the aqueous phase was removed, and the organic phase was concentrated under a stream of nitrogen gas. The same procedure was used to isolate the pentanal aldol condensates and mixed aldol condensates. An aliquot of each aldol condensation product or mixture was injected directly into GC–MS in splitless mode and into the gas chromatography–time of flight (GC–QToF spectrometer).

### Gas Chromatography–Time of Flight

GC–QToF analyses were carried out on an Agilent 7980b, coupled to an Agilent 7200 Accurate Mass Q-TOF in chemical ionization mode (CI) using methane gas (Agilent Technologies, Santa Clara, CA, USA). A nonpolar HP-5MS (30 m × 0.25 mm × 0.25 μm film thickness, Restek, London, UK) column was used. A 1 μL aliquot of synthesized compounds/mixtures diluted in diethyl ether (1:100 synthesized compound:diethyl ether) was injected in splitless mode.

### Nuclear Magnetic Resonance Spectroscopy

To confirm the structure and stereochemistry of the synthesized mixture of stereoisomers, ^1^H nuclear magnetic resonance (NMR) and ^13^C NMR were first carried out on a 400 MHz Bruker Avance III Spectrometer (9.40T) at room temperature (24 °C), followed by nuclear Overhauser effect spectroscopy (NOESY) and correlation spectroscopy (COSY) on a 500 MHz Bruker Avance III Spectrometer (11.75T) with the standard Bruker noesypr1d pulse sequence.

### Quantitation

(*E*)-2-Butyl-2-octenal in the original mix was quantitated by the GC-FID using an external calibration curve constructed using the authentic standard of (*E*)-2-butyl-2-octenal (3–96 μg L^–1^) taking into account the purity as determined by the GC-FID (85%). The concentrations of (*E*)-2-propyl-2-heptenal, (*E*)-2-propyl-2-octenal, and (*E*)-2-butyl-2-heptenal were estimated by using the same external calibration curve.

### Informal Sensory Evaluation

To assess the character of (*E*)-2-butyl-2-octenal at different concentrations, an informal sensory evaluation was carried out. Approval for the study was obtained from the School Research Ethics Committee, study number 31/2024. A panel of 4 experienced assessors collected and discussed odor descriptors at different concentrations, arriving at a consensus on the 2 distinct and different descriptors which they agreed could be labeled as orange and old soap. These terms were then used for informal sensory evaluation. Each panelist was presented with 5 solutions of varying concentration (10%, 1%, 0.1%, 0.01%, and 0.001%) in propylene glycol in random order, with each solution randomized with a 3-digit code. These concentrations were selected based on previous GC–O analysis. The panelists were asked to assess the odor character of the solution. The samples were provided at 1 h intervals because earlier observations had indicated that the assessors might be prone to habituation with the smell. This was repeated on two separate days.

### Threshold Determination

Threshold determination was carried out via GC–O using the synthesized mix of (*E*)-2-propyl-2-heptenal (615 μL/L), (*Z*)-2-propyl-2-heptenal (66 μL/L), (*E*)-2-propyl-2-octenal (41 μL/L), (*E*)-2-butyl-2-heptenal (49 μL/L), (*E*)-2-butyl-2-octenal (49 μL/L), and (*Z*)-2-butyl-2-octenal (8 μL/L). The sample was diluted by using 1 mL of the sample and 2 mL of diethyl ether. The series of compounds in the mix was then assessed by 3 participants in duplicate via GC–O using direct injection (1 μL), until each participant could no longer detect the compound. The same procedure was used for (*E*)-2-decenal. The same GC–O conditions were used as for the SPME samples, except the oven ramp rate was set at 4 °C min^–1^ to ensure good separation of the compounds. The lowest concentration at which the individual assessor detected each of the compounds was recorded. The corresponding dilution factors were calculated and used to approximate the thresholds of each compound for each assessor.

To calculate the individual thresholds of (*E*)-2-propyl-2-heptenal, (*E*)-2-propyl-2-octenal, (*E*)-2-butyl-2-heptenal, and (*E*)-2-butyl-2-octenal in air, the method described by Ullrich and Grosch was used. This method involves the use of a known amount of standard (*E*)-2-decenal and known amounts of each aldol condensation product. The approximation of the individual odor threshold (O_C_) was determined by using the formula below
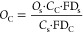
where *O*_C_: odor threshold in air of aldol condensate, *O*_s_: odor threshold in air of standard (2.7 ng L^–1^ as determined by Boelens and Van Germet^19^), *C*_c_: initial concentration of aldol condensate, *C*_s_: initial concentration of standard, FD_c_: dilution factor of aldol condensate, and FD_s_: dilution factor of standard. Thresholds in water were approximated using an experimentally derived linear relationship between thresholds in air and water for *trans*-2-alkenals^19^
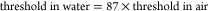


### Statistical Analysis

Averages, standard deviations, and *t* tests were carried out using RStudio version 2024.04.0 + 735 (Posit, Boston, USA). Each sensory evaluation was carried out in duplicate, and analytical tests were carried out in triplicate with a significance level of *p* ≤ 0.05.

## Results and Discussion

### GC–O Analysis of Pea Protein Isolate

The identities of the odor-active compounds, detected in pea protein isolate by up to 6 assessors on a DB-5 column, were confirmed using 3 assessors on a Stabilwax column (Table 1). Compounds with a detection frequency of ≥4 were included in Table 1. A further 3 were included due to their similarity to the old soap odor. In general, the main odors from pea protein isolate were green, grassy, earthy, and beany which matches with other studies on pea protein isolate.^20^ In agreement with previous studies, hexanal was scored the highest among the odor-active compounds.^13,20^ Other frequently detected compounds included 3-methyl-2-butene-1-thiol (marijuana), 1-octen-3-one (mushroom), (*E*)-2-octenal (fatty, green, and medicinal), and 2-nonanone (fruity and woody).

**Table 1:** Odor-Active Compounds in Pea Protein Isolate (PPI)

compound	description^a^	LRI_DB-5_^b^				LRI_Stabilwax_^c^				
		DF^d^ DB5	GC–O DB5	GC–MS DB5	Auth DB5	GC–O wax	GC–MS wax	Auth wax	ID^e^	
hexanal	green, grass	6	798	806	802	1085	1099	1091	O, LRI, MS	
3-methyl-2-butene-1-thiol	marijuana	6	820	821	820	n.d	n.d	NA	O, LRI	
methional	potato^g^	4	901	n.d	912	1468	n.d	1454	O, LRI	
1-octen-3-one	mushroom	5	982	987	983	1271	1270	1302	O, LRI, MS	
dimethyl trisulfide	garlic, mushroom	4	987	975	984	1329	1376	1390	O, LRI, MS	
2-ethyl-1-hexanol	fatty, green	4	1025	1032	1037	n.d	1516	1485	O, LRI, MS	
(E)-2-octenal	fatty, green, medicinal	5	1058	1060	1063	1439	1436	1447	O, LRI, MS	
1-octanol	musty, fruity	4	1079	1074	1073	1560	1564	1542	O, LRI, MS	
acetophenone	sweet, woody	4	1062	1063	1076	1672	1660	1685	O, LRI, MS	
2-nonanone	fruity, woody	5	1094	1093	1092	1388	1392	1375	O, LRI, MS	
nonanal	orange, fruity	4	1110	1105	1107	1398	1396	1391	O, LRI, MS	
(*E*)-2-propyl-2-heptenal	musty, wet soil	1	1192	1196	1196^f^	1476	1477	1477^f^	O, LRI, MS	
(*E*)-2-propyl-2-octenal^g^	old soap, floral	1	1284	1280	1278^h^	1572	1572	1565^i^	O, lri, MS	
(*E*)-2-butyl-2-heptenal^g^	old soap, sweet, floral	2	1296	1293	1293^h^	1587	1587	1587^h^	O, lri, MS	
(*E*)-2-butyl-2-octenal	old soap, green, floral	4	1385	1381	1389^f^	1675	1676	1676^f^	O, LRI, MS	

a Odor descriptors given by 6 experienced assessors.

b Linear retention index on the DB-5 column (n.d.—not detected).

c Linear retention index on the Stabilwax column (n.d.—not detected).

d DF = detection frequency, number of times the odor was detected by assessors (maximum score n = 6).

e Confirmation of identity, where O = odor descriptor agrees with authentic compound, LRI = linear retention index on the DB-5 and/or Stabilwax column agrees with authentic compounds, lri = linear retention index on the DB-5 and/or Stabilwax column agrees with the literature, MS = mass spectrum matches that of the authentic standard (NA—not applicable), ms = mass spectrum agrees with the literature.

f Synthesized standard, ID confirmed by ^1^HNMR.

g Tentative assignment of isomers.

h Synthesized standard.

i Lit value from Buchecker et al.^26^

### Identification of 2-Butyl-2-octenal as a Source of the Old Soap Note

In a previous study by Zhogoleva et al., an unidentified aroma with a LRI on a DB-5 column of 1387 was found and described as a soil and musty odor in pea-based products extracted via SPME.^21^ In our study, a similar odor was found in a pea protein isolate with a similar LRI of 1385 on the same column. We tentatively attributed this odor to the presence of 2-butyl-2-octenal; however, in previous studies, a very wide range of odor descriptors had been used to describe this compound including a sweet citrus odor,^22−25^ as well as grassy, savory, meaty,^22^ old soap,^26^^,^ and cured ham.^27^ 2-Butyl-2-octenal also has some associated unpleasant odors, such as sweaty, oily,^28^ and metallic^23^ (Table 2). To confirm the identity of this odor, an authentic standard of 2-butyl-2-octenal was purchased.

**Table 2:** Odor Descriptors of (E)-2-Butyl-2-octenal

descriptor	origin	citation
green, orange peel, soapy	Valencia orange oil	Abreu et al., 2017
sweetish, metallic, citrus	Scots pine	Schreiner et al., 2018
citrus, grassy, and fruity	Longjing tea	Cheng et al., 2008
cardboard-like, citrus-like, soapy	vehicle interior air	Buchecker et al., 2022
old soap, cardboard, soil, citrus peel	plant-based protein isolates	
green, fruity, metallic, oily, tropical, fatty, sweaty, goaty	authentic standard	TGSC, 2021

The initial odor from the bottle was a weak citrus, orange-rind smell, which was not the distinctly different old soap note that was expected from the GC–O studies. This sample was assessed by GC–O (*N* = 2) at 10 μL/L in diethyl ether using a slower ramp (2 °C min^-1^) to ensure that a good separation was achieved between the *E* and *Z* isomers. The difference in retention time was ∼30 s, corresponding to 12 LRI units. The old soap aroma matched the major isomer of 2-butyl-2-octenal, and no odor was detected corresponding to the minor isomer, indicating that the minor isomer did not provide either the old soap note or the citrus note at the concentration tested. However, when a higher concentration of 2-butyl-2-octenal (1% in diethyl ether) was assessed via GC–O, an orange odor eluted at the same LRI as the major isomer; no old soap was detected, and still no odor was detected for the minor isomer. This was repeated on a polar column with a difference of ∼25 s, corresponding to 11 LRI units. By comparing the LRIs on 2 columns using mass spectrometry, the FID, and odor at the GC odor port, we can confirm that for the two assessors, the major isomer imparted an orange odor at high concentrations and an old soap odor at low concentrations.

### Informal Sensory Evaluation of 2-Butyl-2-octenal

An informal sensory assessment was carried out using 4 assessors to further test our hypothesis that the character of the major isomer of 2-butyl-2-octenal changed with concentration. Participants 1 and 2 consistently described the stronger concentration as orange and the weaker concentration as an old soap odor, which agrees with previous work.^25^ However, different relationships between the concentration and odor type were demonstrated by participants 3 and 4. Participant 3 perceived a mix of odor characters at all concentrations presented, and participant 4 only perceived the presence of a single orange odor at all concentrations. This shows the variation in odor perception between individuals. Participant 4 was able to detect only the orange odor, despite prior training, and was one of the GC–O assessors that was not able to detect the old soap odor in the PPI extract. One hypothesis as to why the odor character changes is the activation by 2-butyl-2-octenal of different odor receptors (ORs).^29^ We suggest that 2-butyl-2-octenal interacts with at least two ORs^30^ which have different activation thresholds, and these also vary between individuals.

### Identification of Other Aldol Condensates as a Potential Source of the Old Soapy Note

In addition to 2-butyl-2-octenal, other uncharacterized old soap notes detected in pea protein (Table 4) were tentatively identified as other aldol condensates and synthesized from single and mixed aldol condensation reactions of pentanal and hexanal. Their presence was verified in the sample using GC–MS. However, Table 1 shows a consistent difference in DB-5 of minus 3–4 LRI units between GC–MS and GC–O which, in our opinion, is too far apart to be absolutely certain that the aromas do indeed match the compound. For this reason, we confirmed that in fact when the same synthesized compounds were injected into the GC–O/FID, excellent matches were obtained (Table 4).

**Table 3:** Mass Spectral Data for (*E*)-2-Propyl-2-heptenal, (*Z*)-2-Propyl-2-heptenal, (*E*)-2-Propyl-2-octenal, (*E*)-2-Butyl-2-heptenal, (*E*)-2-Butyl-2-octenal, and (*Z*)-2-Butyl-2-octenal

compound	isomer	LRI DB-5^a^	LRI wax^b^	mass spectral data, *m*/*z* (relative intensity)^c^	experimental^d^ (theoretical) mass (M + H^+^)
2-propyl-2-heptenal	*E*	1190	1473	55(100), 41.05(95), **154**(**69)**, 125.05(66), 82.95(58), 43(56), 81(48), 39(39), 110.95(37), 29.1(36)	155.1427 (155.1430)
	*Z*	1199	1478	32(100), 55(73), 41(68), 43(58), 83(44), 125(41), 29.1(40), **154**(**32)**, 39(30), 81(29)	155.1420 (155.1430)
2-propyl-2-octenal	*E*^e^	1283	1564	55(100), 111(87), 41(87), 43(63), **168**(**53)**, 83(47), 81(44), 139.05(36), 39(33), 97(33)	169.1587 (169.1584)
2-butyl-2-heptenal	*E*^e^	1290	1574	55(100), 41(95), 125.05(87), 83(63), 29.1(50), 43(49), **168**(**47)**, 97(46), 95(42), 39(34)	169.1581 (169.1584)
2-butyl-2-octenal	*E*	1381	1664	43(57), 139(51), 83(50), 95(46), 29.1(44), **182**(**41)**, 97(40), 125.1(35), 93(35), 69(33)	183.1738 (183.1743)
	*Z*	1393	1673	41(100), 55(92), 29.1(75), 139(61), 43(60), 94.95(48), 82.95(47), 125(41), 79(38), **182**(**35)**	183.1738 (183.1743)

a Linear retention index on DB-5 column.

b Linear retention index on Stabilwax column.

c Molecular ion in bold.

d Experimental mass of compound as determined by GC–QToF.

e Tentative assignment of *E*-isomer based on NMR of the analogues.

**Table 4:** Odor LRI on GC–O and the FID for Aldol Condensates

compound	GC–O	FID (synthesized standard)
(*E*)-2-propyl-2-heptenal (**2**)	1186	1188
(*E*)-2-propyl-2-octenal (**3**)^a^	1285	1284
(*E*)-2-butyl-2-heptenal (**4**)^a^	1292	1291
(*E*)-2-butyl-2-octenal (**1**)	1379	1380

a Tentative assignment of isomer.

**Figure 2:**
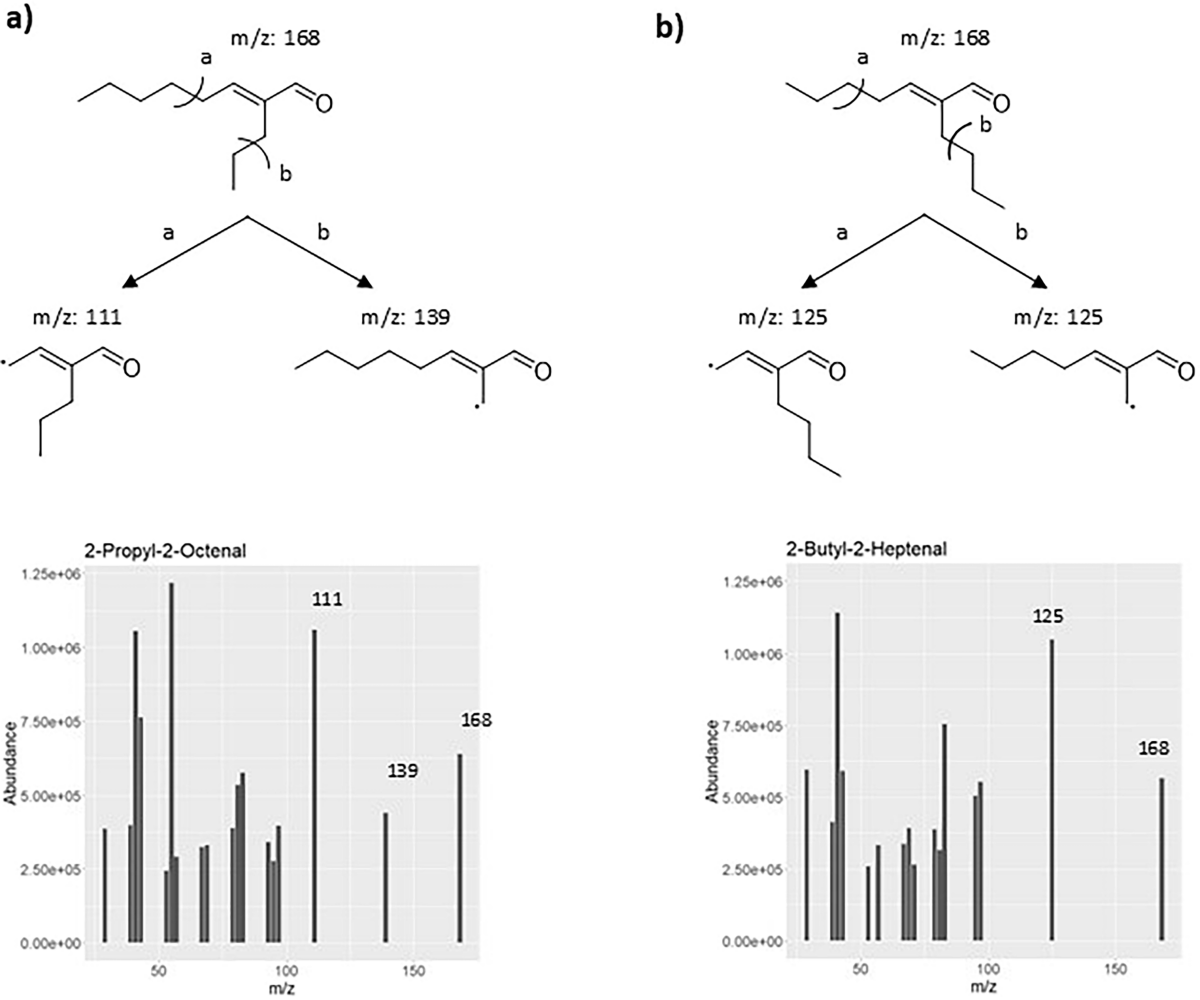
Mass fragmentation patterns of (a) 2-propyl-2-octenal (**3**) and (b) 2-butyl-2-heptenal (**4**), in agreement with Boeswetter et al., 2019.^31^

In plant proteins, the most abundant aliphatic saturated aldehydes (starting materials for this family of aldol condensates) were pentanal and hexanal. From this synthesis, 6 compounds were produced, (*E*)-2-propyl-2-heptenal, (*Z*)-2-propyl-2-heptenal, (*E*)-2-propyl-2-octenal, (*E*)-2-butyl-2-heptenal, and (*E*)-2-butyl-2-octenal and (*Z*)-2-butyl-2-octenal. A library match was found for 2-propyl-2-heptenal,^24^ 2-butyl-2-heptenal,^31^ and 2-butyl-2-octenal;^24^ however, for 2-propyl-2-octenal, there was no library match. (*E*)-2-Propyl-2-heptenal, (*Z*)-2-propyl-2-heptenal, (*E*)-2-butyl-2-octenal, and (*Z*)-2-butyl-2-octenal were fully characterized and their structures confirmed using accurate mass as determined by GC–QToF, 1HNMR, 13C NMR, NOESY, COSY, and high-resolution GC–MS (Table 5). The structures of 2-propyl-2-octenal and 2-butyl-2-heptenal were confirmed by GC–QToF and MS fragmentation (Table 3). 2-Propyl-2-octenal has major fragments at *m*/*z* 111 and 139, whereas 2-butyl-2-heptenal has a major fragment at *m*/*z* at 125 (Figure 2)^31^

### Identification of Isomers

The results of the NMR analysis for 2-propyl-2-heptenal and 2-butyl-2-octenal are listed in Table 5. Here, it is shown that the major isomer (99.5%) for both compounds was the *E* isomer, confirmed by the interaction between the protons at 9.363 ppm (H8) and 6.444 ppm (H6). For the *Z* isomer, 2-propyl-2-heptenal and 2-butyl-2-octenal had no interactions between the protons at 10.120 and 6.858 ppm. The predominance of the E isomer is due to the transition state of the water elimination step favoring the smaller group near the enolate, thus, the two alkyl chains end up in the cis configuration across the double bond. Nomenclature rules dictate that this is the *E* isomer. We therefore propose that the isomers of 2-propyl-2-octenal and 2-butyl-2-heptenal are highly likely to be the *E* isomers, and we have tentatively assigned them as the *E* isomer. Overall, we can confirm that the *E* isomers of aldol condensates formed from hexanal and pentanal are present in PPI and may be responsible for the old soap odors in pea protein isolate.

**Table 5:** NMR Characterization of Synthesized 2-Butyl-2-octenal and 2-Propyl-2-heptenal Isomers

compound	isomer	NMR
2-butyl-2-octenal	*E* (99.5%)	^1^H NMR (CDCl_3_, 400 MHz) 9.362 ppm (s, 1H, H6′), 6.443 ppm (t, *J* = 7.6 Hz, 1H, H8′), 2.358 ppm (q, *J* = 7.2 Hz, 2H), 2.287 ppm (*t*, *J* = 7.2 Hz, 1H), 1.505 ppm (quint, *J* = 7.2 Hz, 3H), 1.340–1.312 (m, 8H), 0.913 ppm (*t*, *J* = 7.2 Hz, 3H), 0.900 ppm (*t*, *J* = 7.2 Hz, 3H) ^13^C NMR (CDCl_3_, 400 MHz) Shift: 195.465, 155.498, 143.821, 31.549, 30.944, 28.888, 28.367, 23.768, 22.763, 22.458, 13.952, 13.900
2-butyl-2-octenal	*Z* (0.5%)	^1^H NMR (CDCl_3_, 400 MHz) 10.120 ppm (s, 1H), 6.860 ppm (*t*, *J* = 7.4 Hz, 1H), 2.358 ppm (q, *J* = 7.2 Hz, 2H), 2.233 ppm (t, *J* = 7.2 Hz, 1H), 1.505 ppm (quint, *J* = 7.2 Hz, 3H), 1.340–1.312 ppm (m, 8H), 0.913 ppm (*t*, *J* = 7.2 Hz, 3H), 0.900 ppm (*t*, *J* = 7.2 Hz, 3H) ^13^C NMR (CDCl_3_, 400 MHz) Shift: 195.465, 155.498, 143.821, 31.549, 30.944, 28.888, 28.367, 23.768, 22.763, 22.458, 13.952, 13.900
2-propyl-2-heptenal	*E* (98.2%)	^1^H NMR (CDCl_3_, 400 MHz) 9.366 ppm (s, 1H, H5′), 6.458 ppm (*t*, *J* = 7.2 Hz, 1H, H7′), 2.37 ppm (q, *J* = 7.2 Hz, 2H), 2.219 ppm (t, *J* = 7.6 Hz, 2H), 1.489 ppm (quint, *J* = 7.6 Hz, 2H), 1.39 ppm (td, *J* = 7.6 Hz, 2H), 1.209 ppm (td, *J* = 7.2 Hz, 2H), 0.941 ppm (*t*, *J* = 7.2 Hz, 3H), 0.896 ppm (*t*, *J* = 7.2 Hz, 3H) ^13^C NMR (CDCl_3_, 400 MHz) Shift: 195.385, 155.58, 143.534, 30.804, 28.661, 25.918, 22.453, 15.228, 14.016, 13.845
2-propyl-2-heptenal	*Z* (1.8%)	^1^H NMR (CDCl_3_, 400 MHz) 10.12 ppm (s, 1H), 6.858 ppm (t, *J* = 7.2 Hz, 1H), 2.37 ppm (q, *J* = 7.2 Hz, 2H), 2.278 ppm (*t*, *J* = 7.6 Hz, 2H), 1.489 ppm (quint, *J* = 7.6 Hz, 2H), 1.39 ppm (td, *J* = 7.6 Hz, 2H), 1.209 ppm (td, *J* = 7.2 Hz, 2H), 0.941 ppm (t, *J* = 7.2 Hz, 3H), 0.896 ppm (t, *J* = 7.2 Hz, 3H) ^13^C NMR (CDCl_3_, 400 MHz) Shift: 195.385, 155.58, 143.534, 30.804, 28.661, 25.918, 22.453, 15.228, 14.016, 13.845

### Threshold Determination

Table 6 shows the lowest concentrations of the sample injected which were detected by each assessor by GC–O. For all assessors, (*E*)-2-butyl-octenal was the most potent of the 4 compounds and (*E*)-2-propyl-heptenal the least. Whereas the lowest concentration of 2-decenal detected was the same for all three assessors, there was a large variation across assessors for the 4 compounds of interest: for example, assessor 2 could detect (*E*)-2-propyl-2-heptenal at a concentration 450 times lower than assessor 3, and assessor 1 could detect (*E*)-2-butyl-octenal at a concentration 700 times lower than assessor 3.

**Table 6:** Lowest Concentration (μg L^–1^) of Aldol Condensates Detected When Injected into GC–O and Approximated Thresholds in Water

compound	assessor 1	assessor 2	assessor 3
lowest concentration injected and detected (μg L^–1^)			
(*E*)-2-butyl-2-octenal (**1**)	0.033	0.30	24
(*E*)-2-propyl-2-heptenal (**2**)	58	3.7	1700
(*E*)-2-propyl-2-octenal (**3**)^a^	6.7	6.7	110
(*E*)-2-butyl-2-heptenal (**4**)^a^	0.88	7.9	130
*(E)*-2-decenal	6.1	6.1	6.1
approximated thresholds in water (μg L^–1^)			
			
(*E*)-2-butyl-2-octenal (**1**)	0.012	0.11	8.7
(*E*)-2-propyl-2-heptenal (**2**)	160	17	7700
(*E*)-2-propyl-2-octenal (**3**)^a^	2.1	2.1	34
(*E*)-2-butyl-2-heptenal (**4**)^a^	0.33	3	49

a Tentative assignment of isomer.

These concentrations were converted to approximate thresholds in water using the equation given in the methods section to calculate thresholds in air, followed by a linear regression based on other long-chain 2-alkenals was used to estimate thresholds in water. The approximate thresholds in water are shown in Table 6. For (E)-2-butyl-2-octenal, assessor 3, who only perceived the orange note, had a similar threshold to the reported value (20 μg L^–1^),^24^ but assessors 1 and 2, who both perceived the old soap note, had thresholds 2000 and 200 times lower, respectively, than the reported value. This is further evidence of the involvement of 2 or more receptors^30^ in the detection of this compound. Assessor 3 may lack the receptor which codes for the soapy note, detecting only the orange note which has a character and odor threshold similar to those of 2-decenal.

### Analysis of Other Protein Isolates

After identification of a family of aldol condensates in pea protein isolate, we investigated their presence in other plant-based proteins and one animal-based protein (WPI) using GC–MS and GC–O. In all cases except two, the aldol condensates which were detected by GC–O were also detected by GC–MS (Table 7). PPI contained significantly more of the most odor-active aldol condensates ((*E*)-2-butyl-2-heptenal and (*E*)-2-butyl-2-octenal) than the other protein isolates, as well as being the only plant protein source to have detectable amounts of (*E*)-2-propyl-2-heptenal. In soy protein isolate (SPI) and rice protein isolate (RPI), the old soap character of (*E*)-2-butyl-2-octenal was detected by GC–O and its presence was confirmed by GC–MS. (*E*)-2-Butyl-2-octenal had been found in soy protein isolate and various varieties of rice in previous studies.^32−37^ Moreover, (*E*)-2-propyl-2-octenal and (*E*)-2-butyl-2-heptenal were found in both SPI and RPI via GC–MS and GC–O. Hemp protein isolate (HPI) contained (*E*)-2-propyl-2-heptenal, (*E*)-2-propyl-2-octenal, and (*E*)-2-butyl-2-octenal, but (*E*)-2-butyl-2-heptenal was not detected. None of the six aldol condensates was detected in whey protein isolate (WPI). This is hypothesized to be due to the isolation technique, as plant proteins use a different method compared to whey protein. Plant protein isolates are commercially isolated by alkaline–isoelectric point precipitation, which uses potassium or sodium hydroxide to increase the pH of hydrated defatted plant-based flour to pH 8–11 to remove insoluble protein fractions and starch fractions.^38^ Moreover, after other pH-altering steps, including adjusting the pH to the isoelectric point of globulin proteins to induce their precipitation, the mixture needs to be dried.^38^ This is most commonly done through spray drying, which applies heat to the isolate. Both the basic conditions and thermal treatment can act as a catalyst for the formation of aldol condensates.^22^ However, whey protein is isolated by using cross-flow membrane filtration, which does not involve the use of strong basic conditions. This suggests that the pH could be a major factor in the formation of these aldol condensates.

**Table 7:** Quantitation (μg/kg) of Aldol Condensates in Protein Isolates and Concentrates Determined by GC–MS Using Dynamic Headspace Extraction^a^

compound	PPI	HPI	RPI	SPI	WPI	PPC 1	PPC 2	YSP
(*E*)-2-propyl-2-heptenal	**16.9±11b**	nd a	**nd a**	nd a	nd a	nd a	nd a	nd a
(*E*)-2-propyl-2-octenal^b^	**25±10bc**	**5.6±6ab**	**21.1±11.6bc**	**9.5±3.4bc**	nd a	nd a	nd a	nd a
(*E*)-2-butyl-2-heptenal^b^	**20.7±5.7c**	**7.0±0.8b**	**11.2±3.3b**	**10.2±6.4bc**	nd a	nd a	**nd a**	nd a
(*E*)-2-butyl-2-octenal	**112.1±16d**	**27.9±4.7b**	**59.7±10c**	**56.5±10c**	nd a	nd a	**49.8±11c**	nd a

a PPI = pea protein isolate, PPC 1 = pea protein concentrate prepared using dry fractionation, PPC 2 = pea protein concentrate with an unknown preparation technique, HPI = hemp protein isolate, RPI = rice protein isolate, SPI = soy protein isolate, YSP = yellow split peas, WPI = whey protein isolate, nd = not detected. Data provided are the average of triplicates; within each row, values with the same letter are not significantly different from each other via the *t* test (*p* < 0.05).

b Tentative assignment of isomer; bolded numbers indicate detection via GC–O analysis.

### Comparison of Pea Protein Isolate and Pea Protein Concentrate

To gain a further understanding of the origin of the aldol condensates in pea protein isolate, 3 other pea-based products were analyzed by GC–MS and GC–O (Table 7): dried yellow split peas (YSP) and two samples of pea protein concentrates: PPC 1 and PPC 2. None of the aldol condensates (**1**–**4**) could be detected in YSP or PPC 1. PPC 1 was prepared using dry fractionation and had not been exposed to the thermal and alkaline conditions involved in isoelectric point precipitation. However, the other pea protein concentrate PPC 2 (from an unknown manufacturing process) contained significant amounts of (*E*)-2-butyl-2-octenal and demonstrated the old soap, cardboard note. This suggests that the preparation of the pea protein extracts might be the cause of the formation of aldol condensates, as this also fits with the known chemistry. It is hypothesized that the use of the base acts as a catalyst for the formation of aldol condensates, as these compounds are formed under basic conditions. In contrast, protein concentrates are typically made by dry fractionation. This process does not involve the use of basic or acidic solvents nor does it involve using thermal treatment.

There are several documented ways to decrease the impact of off-notes in peas, including ethanol washing, fermentation, and heat treatment.^39−41^ For PBMAs, the most popular processing method is extrusion, which involves the use of shear forces and heating. Low-moisture extrusion has been shown to reduce 2-butyl-2-octenal in soy protein isolate and starch using a barrel temperature of 150 °C and moisture level of 20%.^42^ This could be a possible solution for the reduction of aldol condensates; however, many PBMAs are developed using high-moisture extrusion. For improved flavor of PBMAs, we propose a three-pronged approach to mitigating the formation of aldol condensates: (i) reduction of lipid oxidation to reduce the concentration of pentanal and hexanal which are precursors of the aldol condensates, (ii) optimization of the protein extraction process to minimize the aldol condensation, and (iii) optimization of the extrusion parameters to degrade these compounds during extrusion.

It is well-known that lipid degradation is one of the main causes of off-notes in plant-based proteins, but in this paper, we have identified a new family of compounds, derived from common lipid oxidation products such as hexanal and pentanal, which are highly potent and contribute to the off-note. Some consumers may be more sensitive to these notes than others. These compounds were found in several plant protein isolates and are likely to be generated during the alkaline–isoelectric point precipitation used for the manufacture of these isolates. Understanding the source of these off-notes is important for developing better tasting PBMAs that provide a healthy and sustainable alternative to animal-based proteins.
